# Design of a Real-Time and Continua-Based Framework for Care Guideline Recommendations

**DOI:** 10.3390/ijerph110404262

**Published:** 2014-04-16

**Authors:** Yu-Feng Lin, Hsin-Han Shie, Yi-Ching Yang, Vincent S. Tseng

**Affiliations:** 1 Department of Computer Science and Information Engineering, National Cheng Kung University, No.1, University Road, Tainan City 701, Taiwan; E-Mail: aorborcord@idb.csie.ncku.edu.tw; 2 Institute of Medical Informatics, National Cheng Kung University, Tainan City 701, Taiwan; E-Mail: bmhiamso1@idb.csie.ncku.edu.tw; 3 Department of Family Medicine, National Cheng Kung University Hospital, No.138, Sheng Li Road, Tainan City 704, Taiwan; E-Mail: yiching@mail.ncku.edu.tw

**Keywords:** telehealth, care guideline recommendation, continua health alliance, episode mining, personalized care

## Abstract

Telehealth is an important issue in the medical and healthcare domains. Although a number of systems have been developed to meet the demands of emerging telehealth services, the following problems still remain to be addressed: (1) most systems do not monitor/predict the vital signs states so that they are able to send alarms to caregivers in real-time; (2) most systems do not focus on reducing the amount of work that caregivers need to do, and provide patients with remote care; and (3) most systems do not recommend guidelines for caregivers. This study thus proposes a framework for a real-time and Continua-based Care Guideline Recommendation System (*Cagurs*) which utilizes mobile device platforms to provide caregivers of chronic patients with real-time care guideline recommendations, and that enables vital signs data to be transmitted between different devices automatically, using the Continua standard. Moreover, the proposed system adopts the episode mining approach to monitor/predict anomalous conditions of patients, and then offers related recommended care guidelines to caregivers so that they can offer preventive care in a timely manner.

## 1. Introduction

Like many other countries, Taiwan has an aging population, with the elderly (those older than 65) accounting for more than 10 percent of the population in 2010, and this figure expected to increase to more than 15 percent by 2020 [1]. According to statistics from the Department of Health in Taiwan, chronic illnesses are now the major cause of death, especially among the elderly [2], and this has led to a rise in health-related spending. In order to deal with this growing issue, various telehealth systems have been proposed in order to both provide better care and reduce medical expenses.

Although a number of telehealth systems had been developed in recent years [3,4,5,6,7,8], the following problems still exist with most of them: first, in the vital signs measurement process, because the formats used for data transmission and the profiles of the related communication interfaces are based on different standards, healthcare devices cannot be used in a plug-and-play manner, making it difficult to integrate them in one system. In addition, the complex and varied nature of such devices makes it easy for caregivers (in this study, the term caregivers refers to “health care provider” or “hospital nurses” who are trained to provide round-the-clock patient care) to input incorrect data when doing so manually; second, most systems do not focus on reducing the amount of work that caregivers need to do, and provide patients with remote care; third, the alarm functions of existing telehealth systems did not support preventive predictions and do not offer related advice to caregivers. Traditional systems simply send an alarm to caregivers when abnormal vital signs are detected, and the caregivers must then work out how to deal with the situations, which is usually rather inefficient; finally, most telehealth systems were designed for use by patients who are caring for themselves, with few intended for use by caregivers.

In order to address these issues, this study presents a real-time and Continua-based cAre GUideline Recommendation System (abbreviated as *Cagurs*), that can help caregivers to take care of patients, and thus enhance the quality of patient care. The three major advances that *Cagurs* represents are as follows: (1) to make patient care more intelligent, the system not only monitors/predicts the vital signs states so that it is able to send alarms to caregivers in real-time, but also automatically recommends corresponding care guidelines so that appropriate action is taken; (2) to reduce the amount of work that caregivers need to do, and provide patients with remote care, the system can be implemented on mobile devices, based on the caregivers’ requirements and suggestions; (3) vital signs data can be automatically transmitted to the system, and thus there is no need for the caregiver or patient to repeatedly obtain vital signs measurements and manually enter them into the system. 

However, achieving these goals was not an easy task, due to following reasons: first, the formats used for data transmission and the profiles used by the communication interfaces are based on different standards in different healthcare and mobile devices. The functions of plug-and-play, interoperability, and connectivity thus need to be developed in this context; second, data mining algorithms need to be developed in order to produce an effective model that can accurately predict vital signs; third, because the vital signs of patients change over time, the prediction model based on these should be updated in real-time; fourth, the vital signs and physiological changes of interest vary among patients, and so the monitoring and predicting functions of the system need to be personalized for different patients.

To address these issues the current paper proposes a novel telehealth framework for constructing a real-time and Continua-based care guideline recommendation system, known as *Cagurs*. The major contributions of this work are as follows:

- A real-time and personalized vital signs state monitoring/predicting model, called vital signs state predictor (VSP), is provided to predict the vital signs states, give alarms when needed, and recommend related care guidelines to caregivers. In addition, data streaming is also integrated into the VSP model so that it always uses the latest data.
- *Cagurs* can reduce the amount of work that caregivers need to do and can help caregivers improve the efficiency and quality of patient care, and utilizes mobile devices to provide remote care for patients.
- *Cagurs* streamlines the repetitive process of vital signs measurement, while the interoperability and connectivity of the system enables it to save and transmit the vital signs data automatically.
- *Cagurs* has undergone a practical evaluation by caregivers at National Cheng Kung University Hospital, and these users then provides some feedback and suggestions about the system. In addition, the effectiveness of VSP has been demonstrated based on vital signs state predictions made using a publicly available vital signs dataset (obtained from the University of Queensland [9]). The results show that *Cagurs* can successfully deal with the challenging problem of predicting vital signs states.

The reminder of this paper is organized as follows: in Section 2, we review the related works, while Section 3 describes the construction and implementation of *Cagurs*. A number of experiments are described in Section 4. Finally, the conclusions and directions for future works are given in Section 5.

## 2. Related Works

This section reviews the literature on interoperability and connectivity, healthcare using mobile devices, and data mining in a healthcare context.

### 2.1. Continua Health Alliance

The *Continua Health Alliance* (CHA) [10] is a non-profit industry organization with more than 240 participating member companies. The aim of CHA is to establish an ecosystem of interoperable personal connected health systems that lets both individuals and organizations better manage their health and wellness. CHA sets connectivity standards and provides design guidelines that are intended to close recognized interoperability gaps. The design guidelines make use of ISO/IEEE 11073 Personal Health Data [11], and thus help technology developers to build end-to-end, plug-and-play systems more efficiently and cost effectively. The ISO/IEEE 11073-20601 is one of the standards in the ISO/IEEE 11073 standard family, which defines the optimized exchange protocols in conjunction with device specialization standards, and includes the domain information model for data representation, the service model for access definition, and the communication model. The device specializations are the ISO/IEEE 11073-104xx standards which define the requirements for each medical device [12], such as thermometers, pulse oximeters and weight scales.

Bluetooth technology can provide wireless links between devices, and has been widely used to connect healthcare devices for many years. However, the underlying data protocols and formats used with this technology are proprietary, and there is no agreement over the best profile. It is thus necessary for manufacturers to adopt an interoperable wireless standard [13,14,15].

### 2.2. Health Device Profile and Bluetooth Low Energy

*Health Device Profile* (HDP), together with Multi-Channel Adaptation Protocol and Device ID Profile, is a standardized form of Bluetooth communication for medical devices [13]. HDP only defines the mechanism for connection establishment and data exchange, and does not consider the exchange of data between medical devices or the associated data formats. CHA specifies a framework for medical device interoperability and data exchanges based on ISO/IEEE 11073 standards, and medical devices that use the Bluetooth protocol and HDP can refer to this [16]. HDP can be used to carry out channel connection/disconnection, data link creation (reliable or streaming), data link deletion, data link aborting, data link reconnection, data transmission (over one or more data links) and clock synchronization [16]. In 2011, Google Android [17] released BluetoothHealth [18] supporting the ISO/IEEE 11073 standards, and so application developers now do not need to implement HDP, and can employ the Application Programming Interface of HDP to communicate directly with healthcare devices using an Android device. In addition, *Bluetooth Low Energy* (BLE) [19] is a wireless technology which is an open low energy and short range radio technology. In recent years, medical/health devices are designed by considering BLE, and Google Android in Android 4.3 released BLE API [20], supporting the ISO/IEEE 11073 standards, and so application developers now can interact with the BLE devices via the Android BLE API.

### 2.3. Mobile Devices in Patient Care

Some previous studies [4,6] use mobile phones as a terminal to collect users’ vital signs data and provide users guidelines for self-health management. However, because the mobile phones used in these earlier works were not very powerful, most studies used embedded systems [3,5]. Advances in technology mean that in recent years many studies have adopted smartphones or tablets to develop telehealth systems that can help caregivers to provide remote care to patients [21]. However, most of the previous studies reported problems with interoperability between the developed systems and devices, such as blood pressure meters, pulse oximeters, and glucose meters. Because these health devices and systems did not have a common data format, it was difficult for them to exchange information in meaningful way. In 2006, a non-profit industry alliance was formed, called Continua Health Alliance (CHA) [22]. To address the issue of interoperability, CHA has worked to set connectivity standards and the related guidelines. 

### 2.4. Data Mining in Healthcare

Data mining has been applied in many domains such as finance, marketing, and healthcare. Data mining can help users extract useful information from huge amounts of data, thus aiding decision making [10,23]. While data mining has been used to analyze vital signs data [7], such systems have the following problems: first, they cannot handle the huge amount of data that is produced by vital signs monitoring devices; second, the results of data analysis are not directly interpretable by users, and thus the information thus obtained is of limited use. Generally speaking, data mining is used to search anonymous data to establish patterns to apply to the individual. But the vital signs and physiological changes of interest vary among patients, and so the monitoring and predicting functions of the system need to be personalized for different patients. Therefore, we employ episode mining technology to analyze individual medical records. Episode mining is an important data mining technique [24,25,26], which discovers the episodes in an event sequence, with each episode indicating that there is a relationship between events. Although researchers have analyzed vital signs data using episode mining [26], but they do not use this approach to predict the state of vital signs or offer care guideline recommendations. In view of the weaknesses of existing systems, in this work we develop the Continua-based care guidelines recommendation system (*Cagurs*) on mobile devices to assist caregivers. The episode mining technique is applied to build a vital signs state monitoring/predicting model that can send alarms when needed, and also offer related care guidelines.

## 3. Framework Design

**Figure 1:**
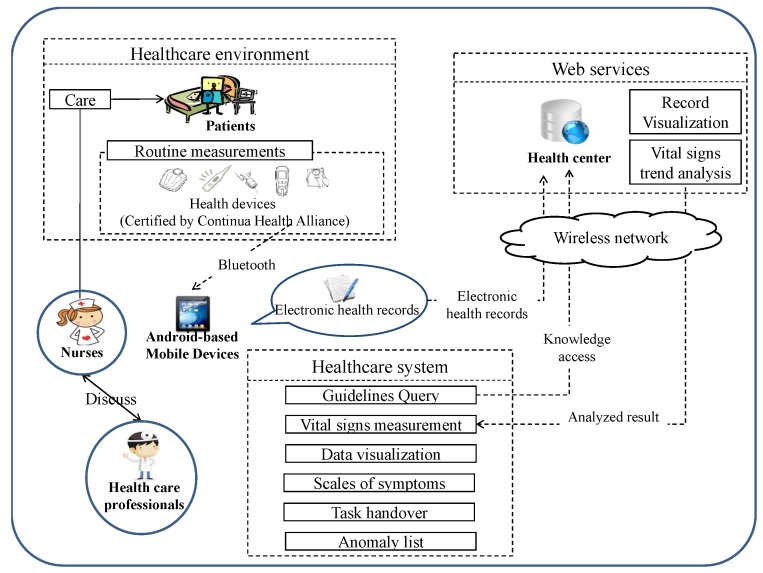
The framework of *Cagurs*.

This section first describes the design of *Cagurs*, based on the framework shown in Figure 1. It then presents an overview of using *Cagurs*, and introduces the functions of the system when implemented on an Android-based mobile device. Finally, we explain the personalized vital signs state prediction model (abbreviated as VSP) used for predicting the vital signs states and offering related guidelines to caregivers.

### 3.1. Overview of System Use

Traditionally, caregivers use paper and pen to record patients’ vital signs data, and then manually input this into the Health Information System (HIS) in Taiwan. If caregivers want to monitor the trend of patients’ vital signs data, or find out the previous vital signs of patients, they have to return to a workstation and perform the related operations. Furthermore, caregivers usually rely on experience to tell whether a patient’s vital signs are normal or not, without the use of any references. This is inconvenient, and can lead to a lower quality of patient care.

To solve these problems, *Cagurs* uses mobile devices with the Health Device Profile (HDP) and adopts ISO/IEEE 11073 standards to automatically transfer the vital signs data on health devices to mobile devices, so that caregivers do not need to return to a workstation to access this information. In addition, the Android application on the mobile devices can connect to a health center using a wireless network, with the center then will transmitting the vital signs states, as well as a prediction of future conditions, in real-time. Here, the format of data transmission is not based on the message format defined by Continua because the *Cargurs* system should be integratable with the HIS systems in different hospitals. If the actual or predicted vital signs states are abnormal, the health center then sends an alarm to the Android application, which shows details of this, as well as related care guidelines, on the screen of the mobile device.

*Cagurs* is designed for patient care, and reducing the amount of work that caregivers need to do in a multidisciplinary in-patient geriatric care ward of hospital, a senior care center, nursing home, a long term care institute, or an elder care and rehab center. For example, a scenario in a multidisciplinary in-patient geriatric care ward is that nurse first uses the health devices to measure vital signs of patients, and the vital signs data is automatically transmitted to mobile devices. Then, the system not only monitors/predicts the vital signs states so that it is able to send alarms to nurse who uses the system in real-time, but also automatically recommends the corresponding care guidelines so that appropriate action is taken. After that, nurse may give the treatment for the patient accordingly, and enter them on the Android-based mobile device. The care/treatment/task can hand over to different shifts of nurses to make sure that the next shift of nurses also can be aware of and understand the previous situations of the patients.

An Android application for mobile devices and a web application for health centers were designed to carry out the goals of our framework. The Android application has the following major functions: it provides a knowledge base of care guidelines, as well as vital signs measurements, data visualization, care/treatment/task handover by different shifts of staffs, scales of Symptoms that are employed to trace the state of certain symptoms and an alarm list. The web application has three major functions: data storage, record visualization and vital signs trend analysis.

### 3.2. Healthcare System

**Figure 2:**
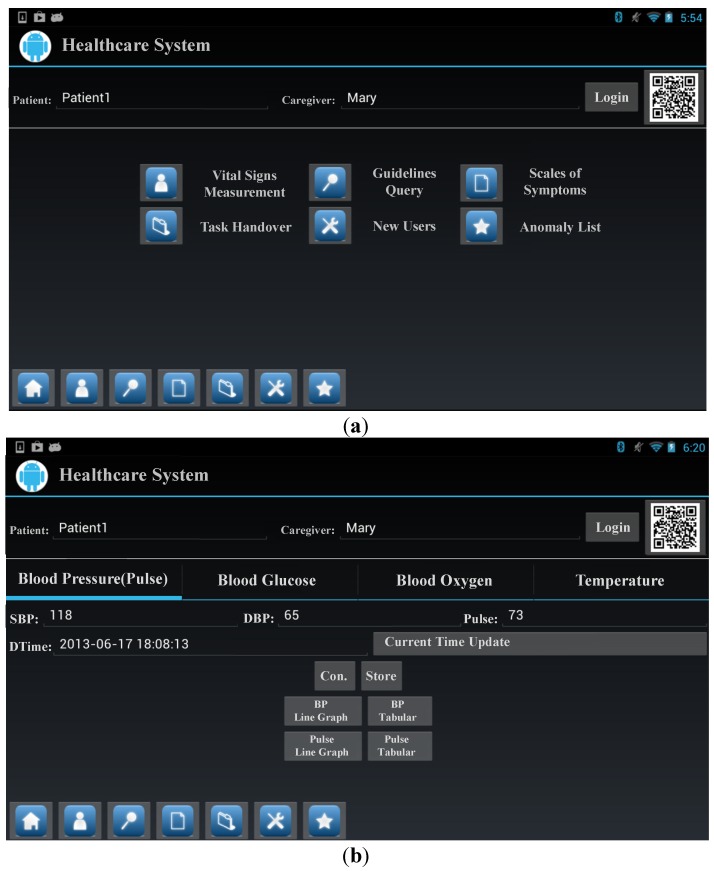
Screenshots of the Android application **: (**a**) User Interface; (**b**) Vital Signs Measurement.

Note: ** While the application was being designed, caregivers suggested that the keywords that are used n it should be the same as those used with the Health Information System (HIS) of the focal hospital, thus making the system easier to use.

This subsection introduces the major functions of the Android application. The user interface of the application has a quick launch bar in order to streamline the procedure when caregivers/users use the Android application. In traditional designs, users generally have to carry out many operations to change functions. For example, users must exit the current function page before turning to another one of interest. To avoid this, we provide a quick launch bar which allows users to directly switch between functions, without repetitive operations, as shown in Figure 2a. In addition, in traditional care procedures, caregivers usually manually record vital signs on paper for each patient. After obtaining these, caregivers then integrate the various sources of data and manually enter them into a computer system. This process is inefficient, and makes it easy for caregivers to mix up the records of patients. We thus provide an automated logging service with QR codes. Caregivers can first enter the patient’s details in the Android application before measuring their vital signs, thus ensuring that the correct data is entered. The use of a QR code automates the procedure of user identification, and maintains the privacy of the patient.

*Vital Signs Measurement***.** *Cagurs* uses HDP, which enables the vital signs measurements to be automatically transmitted from health devices to our system, with the data being transmitted based on the ISO/IEEE 11073 standards. Figure 2b shows screenshots of the vital signs data measurement and how the system automatically collects this information. In addition to saving vital signs automatically, if a sign indicates that there is a problem, by being, for example, outside the boundaries suggested by the Department of Health, then an alarm is sent to users. This alarm not only provides details of the symptom of interest, but also send extra information, such as a definition of the symptom and related care guidelines, and knowledge.

*Data Visualization.* In the Android application, the data visualization function can provide line graphs and tables so that caregivers can easily monitor patients^’^ vital signs. With regard to the line graphs, caregivers can use Two-Point-Touch technology to adjust the axes and time period to see the data more clearly. With the tables, users can set both the vital signs and the time period of interest.

*Guidelines Query***.** The function of guidelines query provides caregivers with an offline database of guidelines, so that they do not need to find a workstation to find this information. In order to meet the caregivers^’^ requirements, the system has a simple rating mechanism which allows users to rate the results returned for each query. This can then help caregivers to more easily find the guidelines they require at their next query.

*Scales of Symptoms***.** Caregivers often employ scales to trace the state of certain symptoms, such as the fall risk assessment scale and geriatric depression scale, with the results then used to decide the correct form of care. The scales of symptoms function in *Cagurs* is designed to enable caregivers to evaluate various symptoms on a mobile device. The system can automatically calculate the sum of the scale and show how serious a patient^’^s symptom is on the screen of the mobile device, with the results being saved to a server so that trends can be seen over time.

*Task Handover***.** Because hospital staffs operate in shifts, a patient may receive care from several people in one day, and thus the issue of care/treatment/task handover is very important. A task handover function is included in the system, so that caregivers can know the history of treatment that each patient has received, and thus adjust their own care plans based on this information.

*Anomaly List***.** The anomaly list shows details of all events that warranted an alarm, such as the related vital signs, when the alarm occurred, and the treatment that was given.

### 3.3. Vital Signs State Predictor

**Figure 3:**
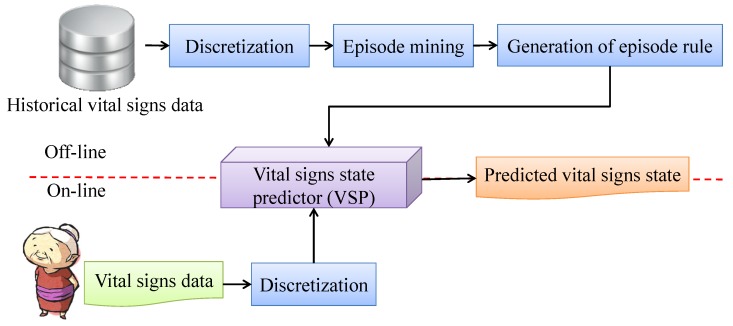
Flowchart of vital signs state predictor (VSP).

This subsection introduces the vital signs state predictor (VSP), with a related flowchart shown in Figure 3. In the offline phase, we discover the episodes from historical vital signs data and generate the episode rules to build the VSP. After this, the vital signs of a patient can be predicted by the VSP. Then, we shall describe in detail the different processes involved in building the VSP.

#### 3.3.1. Discretization

For data discretization, we adopt the standards defined by the Department of Health [2] as reference to transform each vital signs data into events in order to generate a complex event sequence, with the standards shown in Table 1. In brief, an event sequence is a long sequence of events, where each event is described based on the type of symptom, and associated with a time of occurrence.

#### 3.3.2. Mining Frequent Episodes

The aim of mining frequent episodes is at discovering all the episodes with support count no less than a user-specified minimum support count threshold. In our study, we employ the Position Pairs Set (PPS) algorithm to find frequent episodes using minimal occurrences in a complex event sequence [27]. An episode is a set of partially ordered events, and a frequent episode is one that often appears in an event sequence. PPS is used because it is an efficient algorithm, which mines frequent episodes without generating candidates, and can carry out an iterative mining process of full sequence scanning.

To enhance the efficiency of this process, we adopt a tree structure for maintaining the important information of the event sequences related to frequent episodes [25]. The nodes in the tree structure consist of one root labeled as “*root*”, internal node and leaf node. Each internal node registers the label of event, and the root to an internal node forms a simultaneous event prefix. The leaf node registers a set of time points where the corresponding simultaneous event sets occur. We also employ a header table for facilitating the traversal of tree structure, and each entry in header table consists of a time point and a link. The link points to the leaf node which has the same time point as the entry in the tree structure. The tree structure can be constructed during the first scan of complex event sequence. At the same time, the link between header table and tree structure are created. Events in the complex event sequence are rearranged in a fixed order such as lexicographic order.

**Figure 4:**
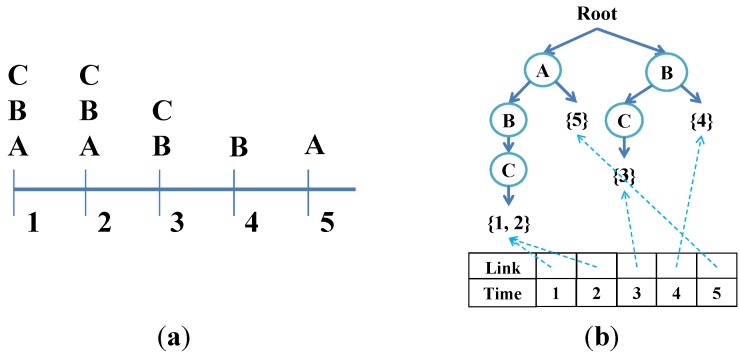
An example tree structure. (**a**) An example of event sequence; (**b**) An example of tree structure.

For example, as shown in Figure 4, we consider the complex event sequence in Figure 4a, when the events at time point 1 are retrieved, three internal nodes corresponding to the events “A”, “B”, and “C” are created. Because the events occur at the time point 1, a leaf node labeled with “{1}” is created. After retrieving all simultaneous events in complex event sequences, the tree structure is constructed completely.

**Table 1:** The blood pressure, oxygen, and heart rate standards.

Blood Pressure (mmHg)		
	Systolic	Diastolic
Prehypertension	120–139	80–89
Stage 1 hypertension	140–159	90–99
Stage 2 hypertension	≥160	≥100
**Oxygen (%)**		
Mild hypoxemia	<94%	
Moderate hypoxemia	<89%	
Severe hypoxemia	<75%	
**Heart Rate (bpm)**		
Tachycardia	>100	
Bradycardia	<60	

Therefore, the events which occur at the same time point are arranged in alphabetical order and then extended from the root, but only the last node records an occurrence. If the path of events already exists, we update the occurrence information or extend it directly, making data storage more efficient. When mining frequent episodes, the events that follow a specific prefix (prefixes and suffixes of episode are subsets of the episode that are included to the beginning or end of the episode. For example, let ABC be an episode, the prefixes of ABC are A and AB; on the other hand, the suffixes of ABC are C and BC) of episode could be found directly by using this tree.

#### 3.3.3. The Vital Signs State Predictor

We generate the episode rules from the frequent episodes that are discovered using episode mining [24], and use these rules to build the vital signs state predictor (VSP). A frequent episode rule is an implication with the form X→Y, where X and Y are events. The rules are ordered by confidence and support that are parameters produced from episode mining processes [25], and the rules with the highest confidence are the ones that are used first to match the incoming vital signs data. If the incoming vital sign is matched by the VSP, the matched results are regarded the future vital sign state of the patient, and these are submitted to healthcare system on Android-based mobile devices. The matching mechanism is designed based on the concept proposed by Chen *et al.* [28].

**Figure 5:**
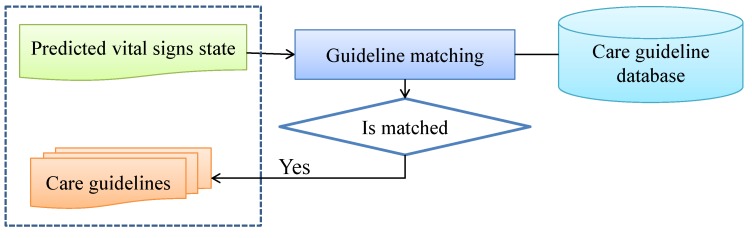
The flowchart of care guideline recommendation.

The predicted vital signs states are then mapped to the related care guidelines, which are sent to the caregivers. If related guidelines are not available, then the system only shows the predicted vital signs state to the caregivers. The flowchart of care guideline recommendation is shown in Figure 5.

#### 3.3.4. Dynamic Updating of the VSP

The vital signs of patients are affected by several factors over time, and these changes are of interest to caregivers. For example, the physiological changes that occur following treatment are very important, and need to be monitored frequently, while the data obtained before treatment is less important. The VSP model is thus based on the vital signs in certain time intervals, and a simple way to achieve this is to employ an efficient episode mining algorithm to rebuild the VSP model when the new vital signs arrive in the system. However, this is a potentially inefficient and expensive process. Therefore, we incorporate the concept of mining data streams into episode mining and propose some strategies to efficiently update the VSP model in real-time, as discussed below.

**Figure 6:**
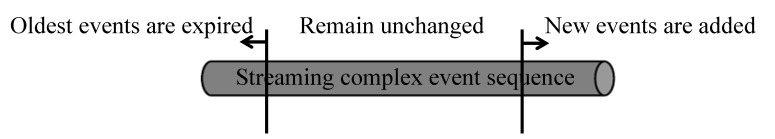
An example of events deletion and addition.

We adopt a sliding-window model to mine episodes from streaming data. Users can set a time interval, and then only the data during this is considered. As Figure 6 shows, when new events are incoming the oldest events are discarded, while the rest of the events remains unchanged. The processes of events deletion and addition are explained in more detail below.

♦ *Events deletion*

The events deletion process discards events which are out of the window. We first discard the episodes using minimal occurrences of the oldest event, and then check if it is frequent. If the current event is frequent, we update the episodes using minimal occurrences of its super-episodes (for example, given three episodes ABC, BC, and B, B and BC are super-episodes of episode ABC. In addition, B is a super-episode of episode BC); if not, we discard all super-episodes of the current event.

♦ *Events addition*

In events addition, we first update the minimal occurrence of the current event, and then two situations are considered. The first is that the new event is frequent in the previous database, and we check all the episodes for which the suffix is the current event, and update their minimal occurrences as needed. The second is that the new event is infrequent in the previous time interval, but now is frequent after updating its minimal occurrence. In this situation, we use temporal join to generate new episodes which are related to the current event [6].

For example, using the event sequence in Figure 4a, assume a user-specified minimum support count = 2, time interval = 4, as well as oldest events and new events are time point 1 and 5, respectively. For the first sliding-window (*i.e.*, time point 1 to 4), the frequent episodes are A:2, B:4, C:3, AB:2, BB:3, BC:2, CB:3, CC:2, BBB:2. When new events (event A) are incoming the oldest events (event A, B, and C) are discarded, we first discard the episodes using events deletion, and then the frequent episodes are updated as B:3, C:2, BB:2, CB:2 (and infrequent episodes are A:1, AB:1, BC:1, CC:1, BBB:1) according to the super-episodes of events A, B, and C. Next, in events addition, because the new event A is infrequent in the previous time interval, we update the support count of event A to be 2 and use temporal join to generate new episodes AA:1, BA:1, CA:1, BBA:1, CBA:1. Last, we integrate the episodes generated in events deletion and events addition, and the frequent episodes are updated as A:2, B:3, C:2, BB:2, CB:2.

Finally, the predicted vital signs states will be mapped to the related care guidelines, which are then sent to the caregiver. If there are no related guidelines, then the system only sends the predicted vital signs state for reference.

## 4. Experiments and Evaluation

To evaluate the novel telehealth framework developed in this study, a practical evaluation was conducted. We implemented *Cagurs* on health devices (FORA D40 blood glucose and blood pressure monitor and Nonin No. 9560 Pulse Oximeter) and a mobile device (Google Nexus 7 running Android 4.1) as the platform for our system. These health devices are certified by Continua Health Alliance, and the measured data are delivered by HDP over Bluetooth. The practical evaluations were carried out by caregivers at National Cheng Kung University Hospital (abbreviated as NCKU hospital) for two separate periods of weeks and one month, after which feedback and suggestions were obtained from the users.

In addition, the effectiveness of the VSP with regard to predicting vital signs was assessed using a public vital signs dataset (obtained from the University of Queensland [9]). This dataset contains vital signs data from 32 anesthesia patients, with the duration of measurement lasting from 13 min to 5 h (median 105 min), with a 10-millisecond sampling rate. The Queensland dataset includes patients undergoing anesthesia for surgery, and the vital signs include data from the electrocardiograph, pulse oximeter, capnograph, noninvasive arterial blood pressure monitor, peak flow meter, and pressure monitor. This work chooses three vital signs data from this dataset, namely pulse oximetry (SpO_2_), Heart Rate (HR), and Arterial Blood Pressure (ABP) to analyze and predict, with these selected as they are usually the most important ones for patients in institutional care.

### 4.1. Practical Evaluation of Cagurs

A clinical trial could not be conducted in this project for the following reasons: (1) *Cagurs* is designed to help caregivers, who are the intended end users of the system, but there is no clinical trial application provided for caregivers; (2) the devices used to measure the vital signs of patients are certified by the Taiwan Medical Device License and Continua Health Alliance, and thus they can be directly used for measurement in patient care, so *Cagurs* is not a new medical technology and a new medical device for treating patients; and (3) because the inpatients only require short stays in hospital, and the collected dataset of vital signs from these patients consist only of limited time series, the dataset is not easy to analyze. In addition, all of the patients taken care of by the same caregiver need to provide informed consent in order to get access to the related data, and caregivers need use different systems if one of their patients does not provide this.

In view of these problems, we conducted a practical evaluation instead of a clinical trial to collect feedback from three caregivers. Each caregiver was given a complete set of apparatus, including a mobile device, blood glucose and blood pressure monitor, and pulse oximeter. A brief tutorial was given on how to use the application, followed by a pre-trial questionnaire to collect their first impressions of the system. In addition, to protect patient privacy the vital signs data that was collected was not retained in the system.

The caregivers reported that the process of automatic vital signs transmission streamlines the vital signs measurement procedure, avoids inputting incorrect data, and ensures the care given is correct. In addition, they stated that the early alarm and care guideline recommendation functions helped them to adjust the care plans for their patients in real-time, and that this was especially helpful for inexperienced caregivers. In addition, they reported that their knowledge about care was improved based on the rules that the system discovered. Moreover, the caregivers said that the size of the Google Nexus 7 device made it very easy to carry, and thus it was convenient to give remote care to patients. The caregivers also made some comments with regard to improving the system, such as making the icons in the user-interface bigger. Additionally, although we provided a convenient way for users to login, the procedure must be repeated before measuring the vital signs from each patient. Finally, the caregivers suggested that the system should include a function to record patients’ drugs, treatments and symptoms combined with vital signs visualization.

### 4.2. Experiments on Real Data

In this subsection we evaluate the performance of the VSP using the Queensland dataset. All experiments were conducted on a computer equipped with an Intel Core 2 Processor (3.40 GHz, 1 GB RAM), running on Windows 7. The discretization standards for each vital sign are shown in Table 1.

**Figure 7:**
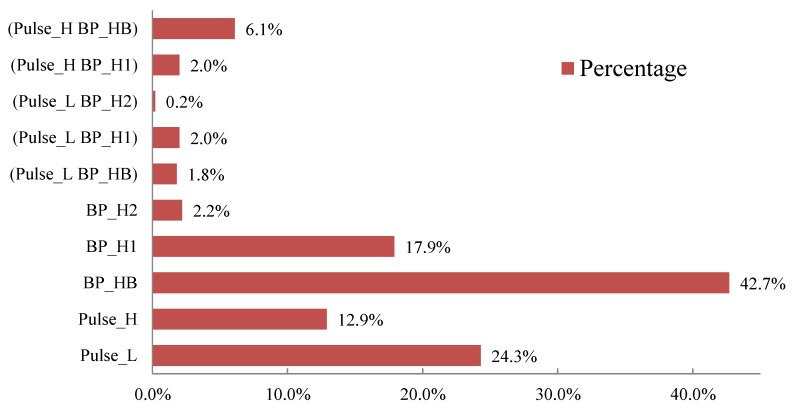
The distributions of vital signs states in the Queensland dataset.

Because different patients have different conditions, the distributions of vital signs states also vary, and this may affect the rule generation results using episode mining. Table 2 shows the abbreviations of the vital signs states, so that they can be more easily represented. In order to explain the results of following experiments, we first analyze the distributions of vital signs states in the Queensland dataset, as shown in Figure 7. Figure 7 considers all the combinations of vital signs presented in Table 2, as follows: pulse_L, pulse_H, BP_HB, BP_H1, BP_H2, (pulse_L BP_HB), (pulse_L BP_H1), (pulse_L BP_H2), (pulse_H BP_H1), and (pulse_H BP_HB). All of these states and combinations were thus used to build the VSP.

**Table 2:** The abbreviations of vital signs states.

Vital Signs	Abbreviation	Vital Signs	Abbreviation
Prehypertension	BP_HB	Severe hypoxemia	SpO2_Lhard
Stage 1 hypertension	BP_H1	Tachycardia	Pulse_H
Stage 2 hypertension	BP_H2	Bradycardia	Pulse_L
Mild hypoxemia	SpO2_Lmicro	Normal	N
Moderate hypoxemia	SpO2_Lmid		

We use precision, recall and F-measure measurements to evaluate the performance of the prediction model [22], and these are defined as follows:

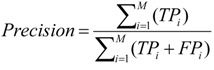
(1)

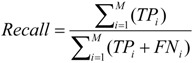
(2)

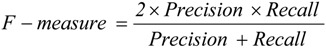
(3)
where M is the number of categories and TP, FP and FN are the numbers of true positives, false positives and false negatives, respectively. A true positive (TP) is when the model predicts the *i*-th class label as “(Prehypertension)” and the *i*-th ground truth class label is “(Prehypertension)”. A false positive (FP) is when the model predicts the *i*-th class label as “(Prehypertension)”, and the *i*-th ground truth label is “(Tachycardia Prehypertension)”. A false negative (FN) is when the model predicts the *i-*th class label as “(Prehypertension)”, and the *i-*th ground truth label is non-“(Prehypertension)”.

**Figure 8:**
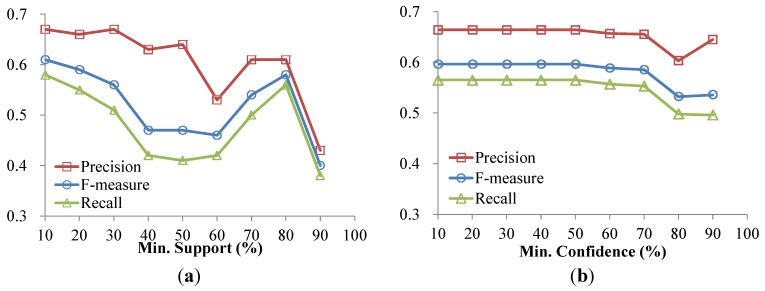
Precision, Recall and F-measure with varying min. support and min. confidence. (**a**) Varying Min. Support; (**b**) Varying Min. Confidence.

The performances under various minimum supports and minimum confidences are shown in Figure 8. Figure 8a shows the different measurements of the prediction models on the Queensland Vital Signs Dataset under various minimum support thresholds when the minimum confidence is set at 70%. As shown in Figure 8a, when the minimum support is set at 10%, the F-measure is more than 0.6 and the precision and recall are also better than at other settings. This because significant rules with high confidence may be generated when the minimum support is set low. Figure 8b shows the different measurements under various minimum confidence thresholds when the minimum support is set at 20%. As seen in Figure 8b, the performance of vital signs state prediction is more than 0.55. Overall, the results show that there is good performance with regard vital signs states prediction in terms of precision, recall and F-measure.

Finally, we collected the rules discovered from the University of Queensland Vital Signs Dataset and discussed them with medical professionals at NCKU Hospital. It is worth mentioning that some of the rules have physiological meaning, based on the opinions of the medical professionals, as shown in Table 3, Common Rules. In addition, we also discovered that some of the rules from the Queensland dataset only occur under certain conditions. For example, rule “(Prehypertension) → (Tachycardia Prehypertension)” only happens when the body of anesthesia patient has been injured or during hemorrhage.

**Table 3:** Discovered rules.

Common Rules
(Tachycardia Prehypertension) → Prehypertension
(Tachycardia), (Prehypertension) → Prehypertension
(Hypertension_I) → (Hypertension_I)
(Tachycardia) → (Prehypertension)
(Tachycardia), (Tachycardia) → (Prehypertension)
(Tachycardia) → (Tachycardia Prehypertension)
**Conditional Rules**
(Prehypertension) → (Tachycardia)
(Prehypertension) → (Tachycardia, Prehypertension)

## 5. Conclusions and Future Work

This work presented a novel telehealth framework based on Continua Health Alliance standards in order to provide better patient care with the support of care guidelines recommendation service on Android-based mobile devices. The implemented system was subjected to a practical evaluation by caregivers at NCKU Hospital, who also offered some feedback and suggestions about the system. The feedback from the caregivers showed that the proposed system can streamline the otherwise repetitive process of vital signs measurement, aid the care procedure, and raise the quality of patient care. In addition, this work also used episode mining to build a vital signs state monitoring/predicting model so that users can be alerted when any abnormalities occur, and related care guidelines can also be provided. The experimental results show that the vital signs state prediction model has good performance, and can discover rules which have physiological meaning. Overall, we have proven that *Cagurs* is feasible for use in a clinical environment.

With regard to future developments, we consider collecting more patient information for use by the system, such as data on the drugs and treatments that they have been given. In addition, we would like to apply our framework to other scenarios, such as continually monitoring vital signs data in intensive care units and long-term care institutions.

## References

[1] Council for Economic Planning and Development. http://www.ndc.gov.tw/encontent/.

[2] Department of Health, Executive Yuan R.O.C. http://www.mohw.gov.tw/.

[3] Chen M.J., Chen K.Y., Chiang S.J., Chang P. Taipei Smart Medical Package. Proceedings of the 14th IEEE International Conference on e-Health Networking, Applications and Services (Healthcom).

[4] Farmer A., Gibson O., Hayton P., Bryden K., Dudley C., Neil A., Tarassenko L. (2005). A real-time, mobile phone-based telemedicine system to support young adults with type 1 diabetes. Inform. Prim. Care.

[5] Lim J.H., Park C., Park S.J. Home Healthcare Settop-box for Senior Chronic Care Using ISO/IEEE 11073 PHD Standard. Proceedings of the Annual International Conference of the IEEE Engineering in Medicine and Biology Society.

[6] Matsumoto T., Ogata S., Kawaji S. Designing and Implementation of Mobile Terminal for Telehealth Care Life Support System. Proceedings of the 7th International Conference on Computer Supported Cooperative Work in Design.

[7] Tsai H., Lin Y.F., Yang Y.C., Tseng V.S. A Mobile Framework for Personalized Diabetes Telecare. Proceedings of the Conference on Technologies and Applications of Artificial Intelligence.

[8] Su C.-J., Chiang C.-Y. (2013). IAServ: An intelligent home care web services platform in a cloud for aging-in-place. Int. J. Environ. Res. Public Health.

[9] Liu D., Gorges M., Jenkins S.A. (2011). University of Queensland vital signs dataset: Development of an accessible repository of anesthesia patient monitoring data for research. Anesth. Analg..

[10] Continua Health Alliance. http://www.continuaalliance.org/products/design-guidelines.

[11] IEEE Standards Association Page. http://standards.ieee.org.

[12] ISO/IEEE 11073 Personal Health Data. http://person.hst.aau.dk/ska/MIE2008/ParalleSessions/PresentationsForDownloads/Mon-1530/Sta-30_Clarke.pdf.

[13] Health Device Profile Implementation Guidance Whitepaper. https://www.bluetooth.org/docman/handlers/downloaddoc.ashx?doc_id=225927.

[14] Park K.H., Pak J.G. (2012). Implementation of a handheld compute engine for personal health devices. Int. J. Smart Home.

[15] Wu J.R., Tsai Y.S. Building a Home Care System with the ISO/IEEE 11073 Standard. Proceedings of the International Federation for Medical and Biological Engineering.

[16] Latuske R. (2009). Bluetooth Health Device Profile and the IEEE 11073 Medical Device Framework.

[17] Google Inc Android Developers. http://www.android.com/.

[18] BluetoothHealth. http://developer.android.com/reference/android/bluetooth/BluetoothHealth.html.

[19] Bluetooth Low Energy. http://www.bluetooth.com/Pages/low-energy-tech-info.aspx.

[20] BLE API. http://developer.android.com/guide/topics/connectivity/bluetooth-le.html.

[21] Patel K., Chapman C.G., Luo N., Woodruff J.N., Arora V.M. (2012). Impact of mobile tablet computers on internal medicine resident efficiency. Arch. Intern. Med..

[22] Yang Y., Liu X. A Re-examination of Text Categorization Methods. Proceedings of the 22nd Annual International ACM SIGIR Conference on Research and Development in Information Retrieval.

[23] Koh H.C., Tan G. (2005). Data mining applications in healthcare. J. Healthc. Inf. Manag..

[24] Mannila H., Toivonen H., Verkamo A.I. (1997). Discovering frequent episodes in sequences. Data Min. Knowl. Discov..

[25] Wu C.-W., Lin Y.-F., Yu P.S., Tseng V.S. Mining High Utility Episodes in Complex Event Sequences. Proceedings of the 19th ACM SIGKDD Conference on Knowledge Discovery and Data Mining.

[26] Patnaik D., Patrick B., Ramakrishnan N., Parida L., Keller B.J., Hanauer D., Hanauer D.A. Experiences with Mining Temporal Event Sequences from Electronic Medical Records: Initial Successes and Some Challenges. Proceedings of the 17th ACM SIGKDD International Conference on Knowledge Discovery and Data Mining.

[27] Ma X., Pang H.H., Tan K.L. Finding Constrained Frequent Episodes Using Minimal Occurrences. Proceedings of the 4th IEEE International Conference on Data Mining.

[28] Cho C.W., Wu Y.H., Yen S.J., Zheng Y., Chen A.L.P. (2011). On-line rule matching for event prediction. VLDB J..

